# The Impact of Chitosan-Divergicin Film on Growth of *Listeria monocytogenes* in Cold-Smoked Salmon

**DOI:** 10.3389/fmicb.2018.02824

**Published:** 2018-11-26

**Authors:** Rajaa Benabbou, Muriel Subirade, Michel Desbiens, Ismail Fliss

**Affiliations:** ^1^Department of Food Science, Faculty of Agriculture and Food Sciences, Institute of Nutrition and Functional Foods, Université Laval, Québec City, QC, Canada; ^2^Centre Technologique des Produits Aquatiques, Ministère de l’Agriculture des Pêcheries et de l’Alimentation, Gaspé, QC, Canada

**Keywords:** *Listeria monocytogenes*, divergicin M35, chitosan, chitosan-divergicin film, cold-smoked wild salmon

## Abstract

The aim of this study was to evaluate the impact of chitosan film, with bacteriocin divergicin 35 incorporate, on growth of *Listeria monocytogenes* in Cold smoked salmon. The simples of Cold-smoked wild salmon were inoculated with *L. monocytogenes* and treated with chitosan (100 kDa, 94.7% de-acetylated) and divergicin M35 was stored for 3 weeks at 4–8°C. The compounds were applied to the fish flesh in the form of solution or dried film. The film reduced *L. monocytogenes* to below the detection limit (<50 cfu/g) and kept total counts below 10^4^ cfu per g compared to 10^9^ cfu per g in control samples while the effectiveness of the solution was very limited. The inhibitory activity of the film lasted for 3 weeks, while the solution had no effect on *L. monocytogenes* counts measured on day 14. The film provided a better preservation of fish color (redness) and firmness than others treatments, while the solution had little impact on these parameters. It kept the volatile basic nitrogen (17.5 mg N/100 g) below the control value 29.9 mg N/100 g. Divergicin-loaded chitosan film thus may represent an interesting alternative for the bio-preservation of cold-smoked fish.

## Introduction

*Listeria monocytogenes* is a foodborne human pathogen responsible for an estimated 28% of food-related deaths each year in the United States (Mead et al., 1999). On August 2008, Ready to eat products were responsible or a multi-state outbreak in Canada that resulted in 57 cases of listeriosis have been confirmed (mostely in Ontario), and 22 confirmed deaths (Public Health Agency of Canada, 2008). Compared to other foodborne pathogens, the mortality rate associated with *L. monocytogenes* infection is relatively high (∼20% compared to <1% for *Salmonella* or *Escherichia coli* O157) (Crerar et al., 1996; De Valk et al., 2005; Scallan et al., 2011). This pathogen is widespread in the environment and can grow in many food products (Alhogail et al., 2016).

Cold-smoked salmon is a good substrate for *L. monocytogenes* growth (Pelroy et al., 1994; Duffes, 1999; Katla et al., 2001; Brillet et al., 2005) because it offers favorable proliferation conditions such as, neutral pH and high water activity, which allow this organism to grow even at refrigeration temperatures. The prevalence of *L. monocytogenes* has been reported in several studies to be as high as 34–43% in cold-smoked salmon (Jorgensen and Huss, 1998). Ben Embarek (1994) reported values between 0 and 75% with an overall prevalence of 10%, and Gombas et al. (2003) reported 4.31% in smoked seafood in Maryland and northern California in 2000 and 2001. The cold smoked salmon contamination with *L. monocytogenes* can be related to several factors such as cleaning and sanitation practices applied during cold smoked salmon production (Jorgensen and Huss, 1998; Johansson et al., 1999) the plant environment, the refrigerated storage and absence of guidance on the fundamental principles of microbiology, including testing for *L. monocytogenes* (Rotariu et al., 2014).

Bio-preservation has been proposed as means of improving food quality and safety. This approach is based on using protective bacterial cultures or antimicrobial compounds obtained there from, in particular bacteriocins, to control spoilage and pathogenic microorganisms such as *L. monocytogenes* (Nilsson et al., 1997; Tahiri et al., 2009). Different strategies have been described for the incorporation of bacteriocins into food matrices. These include direct inoculation with the producing strain, adding purified or semi-purified bacteriocin and immobilizing antimicrobial agents on solid supports such as polymer coatings or films (Fu et al., 2016). This latter approach offers several advantages over the other two, notably better protection of the active compound from inhibitors by decreasing its interaction with the food matrix, maintenance of a high concentration on the food surface, slow and continuous release during food storage and synergistic effects with antimicrobial properties of the polymer support (Coma et al., 2001, 2002; Cutter et al., 2001; Pranoto et al., 2005; Elsabee and Abdou, 2013).

Chitosan is a polymer obtained by deacetylation of chitin obtained from crustacean shells and fungi such as *Aspergillus niger, Mucor rouxii*, and *Penicillium notatum* (Tan et al., 1996; No et al., 2002; Qin et al., 2006). Chitosan is now widely recognized as non-toxic, biocompatible and biodegradable (Ye et al., 2008a). The Japanese department of health classified chitin and its derivatives as functional food ingredients in 1992. More recently (2001), the US FDA approved the GRAS status of chitosan. The capacity of chitosan to form films with excellent mechanical and physicochemical properties (Begin and Van Calsteren, 1999; Srinivasa et al., 2004; Suyatma et al., 2004) and to inhibit a wide variety of microorganisms (Shahidi et al., 1999; Agullo et al., 2003; Rinaudo, 2006; Moreira et al., 2011) makes it an interesting polymer for the development of films as antimicrobial delivery systems. Chitosan films containing added natural antimicrobial agents such as organic acids, essential oils, potassium sorbate, lysostaphin, polyphenols, sodium lactate, and bacteriocins have been shown to inhibit several pathogens in foods (Ouattara et al., 2000; Cha et al., 2003; Pranoto et al., 2005; He et al., 2016; Schelegueda et al., 2016; Nithya et al., 2018). Several studies have demonstrated the antimicrobial effect of chitosan films on pathogens in simple models with pure cultures (Pranoto et al., 2005; Li et al., 2006). A few more publications have used chitosan-base packaging films in food such as meat, poultry and fish (Beverlya et al., 2008; Higueras et al., 2013). The intrinsic activity of low-molecular-weight chitosan alone in combination with added antimicrobial compounds has rarely been investigated. To the best of our knowledge, no study has been published on the feasibility of using chitosan based film containing bacteriocin to improve the preservation of cold-smoked salmon during storage.

Divergicin M35 is a class IIa bacteriocin produced by a strain of *Carnobacterium divergens* M35 isolated by our group from frozen mussels (Tahiri et al., 2004). In a previous study, we showed synergetic inhibition by divergicin M35 and low-molecular-weight chitosan against *L. monocytogenes* LSD532 (Benabbou et al., 2009). A bio-ingredient containing a mixture of *C. divergens* and divergicin M35 has been developed and found to be a strong inhibitor of *Listeria* on smoked salmon. Health Canada recently has approved this bio-ingredient as a new additive for smoked fish (Public Health Agency of Canada, 2016).

The aim of the present study was to evaluate the potential of chitosan-divergicin M35 film as an inhibitor of *L. monocytogenes* on cold-smoked salmon stored for 3 weeks at refrigerator temperatures and the impact of the antimicrobial film on the physical and chemical properties of this seafood product. The impact of chitosan-divergicin M35 film on growth of *L. monocytogenes* was compared with treatment using chitosan-divergicin M35.

## Materials and Methods

### Materials

Chitosan (100 kDa, 94.7% de-acetylated) was obtained from DNP Canada inc. (Granby, QC, Canada). It was provided in 1% (v/v) acetic acid and stored at -20°C until use.

Divergicin M35 was produced and purified using a protocol similar to that previously described by (Tahiri et al., 2004) but adapted for higher culture supernatant volume. Purified divergicin M35 was obtained from supernatant of *C. divergens* M35 MRS culture. Supernatant (500 ml) was heated in a water bath at 100°C for 10 min and then passed through an SP-Sepharose Fast Flow Cation Exchange Column (Amersham, Pharmacia Biotech, Uppsala, Sweden) at flow rate of 3 ml/min. The column was then washed and equilibrated with 1 l of ammonium acetate buffer (5 mM, pH 5). The bacteriocin was eluted with 250 ml of 1.5% (w/v) sodium chloride in ammonium acetate buffer, loaded onto a Sep-Pack C18 Cartridge micro-column (Waters, Milford, MA, United States) previously equilibrated with 5 mM of HCl in HPLC grade water and eluted from the Sep-Pack with 60 ml of 50% (v/v) acetonitrile in water. Acetonitrile was removed using a rotary evaporator. Bacteriocin was concentrated under vacuum with a Speed-Vac concentrator (Thermo Savant Instruments Inc., NY, United States) and kept at -80°C until use. Antimicrobial activity of Divergicin M35 during the steps of purification against *L. innocua* HPB13 was confirmed using the agar diffusion method (Tagg et al., 1976).

*Carnobacterium divergens* M35 was grown in de Man, Rogosa and Sharpe (MRS) broth (De Man et al., 1960) obtained from Rosell Institute (Montreal, PQ, Canada) containing 0.1% (v/v) Tween 80 and incubated aerobically at 30°C. *L. monocytogenes* strain LSD532 (*L. monocytogenes*) was obtained from the Canadian Food Inspection Agency Laboratory Services Division (Ottawa, ON, Canada). *L. innocua* HPB13 (*L. innocua*, used as an indicator) was obtained from Health Protection Branch, Health and Welfare Canada (Ottawa, ON, Canada). Both strains were maintained as 20% glycerol stock at -80°C. *L. innocua* and *L. monocytogenes* were grown aerobically at 30°C and 37°C, respectively, in tryptic soy broth (TSB, Difco Laboratories, Sparks, MD, United States) supplemented with 0.6% (w/v) yeast extract (YE, Difco).

### Film-Forming Solution and Film Preparation

Chitosan-divergicin M35 (C-M35) solution was prepared by blending stock solutions to obtain 0.125 mg/ml divergicin M35 and 6.25 mg/ml chitosan in 1% (v/v) acetic acid.

Chitosan-divergicin M35 film was prepared by pouring 145 ml of solution into Petri dishes to a height of 1 cm and drying the liquid in a laminar flow hood for 72 h at ambient temperature. The resulting film was peeled and used directly for each experiment. Chitosan film was made also without divergicin M35 for comparison.

### Preparation of *L. monocytogenes* Inoculum

*Listeria monocytogenes* was grown in TSBYE at 37°C and cells were harvested by centrifugation at 7,500 × *g* for 15 min at 4°C, washed twice with sterilized phosphate buffer saline: PBS (0.01 M phosphate, pH 7.2) and re-suspended in 10 ml of PBS. Buffer was added to obtain a final concentration of approximately 10^5^ cfu/ml. Dilutions were plated on TSBYE plates and incubated aerobically at 37°C for 24 h before determining viable cell counts.

### Assays With Cold-Smoked Salmon

Cold-smoked wild Pacific sockeye salmon (hereinafter ‘fish’) was provided by Fumoir Grizzly Inc. (St-Augustin, Québec, Canada) and used immediately upon reception. The flesh was cut into squares (3 cm, 4 g) and divided into six groups corresponding to the following treatments:

Treatment A: control (untreated) samples.

Treatment B: 100 μl of *L. monocytogenes* suspension (10^5^ cfu/ml) spread on one side of each square (2.5 × 10^3^ cfu/g).

Treatment C: 100 μl of C-M35 solution spread on the side inoculated with *L. monocytogenes.*

Treatment D: inoculated squares covered with 9 cm^2^ of C-M35 film.

Treatment E: un-inoculated squares coated with 100 μl of C-M35 solution.

Treatment F: un-inoculated squares covered with 9 cm^2^ of C-M35 film.

The experiments were performed in duplicate and each simple was analyzed twice. Simples were dried for 10 min in a laminar-flow biological safety cabinet, wrapped in oxygen permeable film obtained from Fumoir Grizzly Inc., and incubated for 21 days under the conditions suggested by AFNOR (Association Française de Normalisation NF V 45-065, 1997): 14 days at 4°C followed by 7 days at 8°C. These conditions are designed to simulate changes in temperature during storage and handling in commercial and domestic environments.

Color, texture and total volatile basic nitrogen (TVBN) were determined at 1, 7, 14, and 21 days of storage. Microbial composition, pH and antimicrobial activity were determined at 1, 3, 7, 14, and 21 days of storage.

### Microbiological Analysis

The whole 4 g sample was placed in a sterile filtering stomacher bag (Seward Medical, London, United Kingdom) with peptone water (0.1% w/v) to obtain a 1/10 dilution (i.e., 36 ml). For treatment D, the C-M35 film was removed first and the films were processed the same way (in 36 ml of peptone water) for measurement of the residual inhibition activity. Bag contents were homogenized for 3 min using a Stomacher 400 circulator (Seward, Therfford, Norfolk, United Kingdom). The filtered homogenate was serially diluted 10-fold in peptone water and dilutions were spread-plated in duplicate on appropriate selective media. For *L. monocytogenes*, CM0856 *Listeria* selective medium supplemented with SR140 *Listeria* selective supplement (Oxoid Ltd., Basingstoke, Hampshire, England) was used and plates were incubated aerobically at 37°C for 48–72 h. Total lactic acid bacteria (LAB) were enumerated on nitrite actidion polymyxin (NAP) agar (Davidson and Cronin, 1973). NAP medium consists of APT agar (Difco Laboratories) supplemented with Polymyxin B (0.003 g/l), cycloheximide (0.01 g/l) and NaNO_2_ (0.6 g/l), all obtained from Sigma-Aldrich (Oakville, Ontario, Canada). Plates were incubated aerobically at 25°C for 48 h. For the determination of the total viable bacterial counts, dilutions were spread-plated on plate count agar (PCA, Difco Laboratories, Sparks, MD, United States) and incubated aerobically at 30°C for 48 h.

### Antimicrobial Activity

Residual inhibitory activity in fish and film in treatments C and D was determined by centrifuging homogenate (7,500 × *g* for 15 min) at 4°C and filtering the supernatant using sterile syringe filters (0.45 μm, Corning, Germany). The agar diffusion method and a quantitative critical dilution micro-method (Tahiri et al., 2004) were used with *L. innocua* HPB13.

### Physical and Chemical Analysis

Color parameters L, ‘a’ and ‘b’ (brightness, redness and yellowness) were measured using a Minolta Chroma Meter CR-300 (Minolta Camera Co., Ltd., Osaka, Japan) with the CIE color system (CIE, 1996). Measurements were made after standardizing the instrument with light source D and the measuring head was rotated 90° between duplicate measurements at each position. Two fish samples per treatment (A, E, and F only) were measured four times each and the mean of the 8 measurements was used for statistical analyses.

Total volatile basic nitrogen (TVBN) production was determined by a distillation procedure (Pearson, 1976) and the pH of the homogenate was measured.

Texture profile analysis (TPA) involved using a Texture Analyzer TA.XT2 (Stable Micro Systems, Texture Technologies Corps, Scarsdale, NY, United States) with double compression. Firmness was defined as the peak force of the first compression and cohesiveness was defined as the ratio of the total energy required for the second compression to that of the first compression. The probe (a flat-ended cylinder 4 mm in diameter) descended at a constant speed of 1 mm/s. Four measurements were performed on each of two samples per treatment and the mean value was used for statistical analyses.

### Statistical Analyses

Statistical analyses were performed based on duplicate and each simple was analyzed twice. Statistical analyses were performed using STATGRAPHICS plus 4.1 (Manugistics Inc., Rockville, MD, United States). Significant differences among the treatment means of each parameter were tested by analysis of variance. Treatment comparisons were performed using Fisher’s least-significant differences (LSD) test with a *P*-value of ≤0.05 considered significant.

## Results

### Microbiological Analysis

**FIGURE 1:**
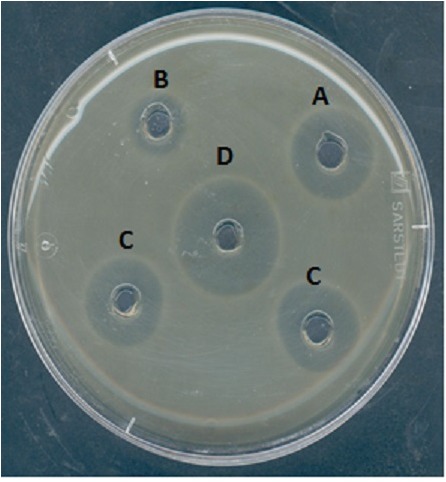
Inhibition of *Listeria innocua* HPB13 by divergicin M35 from *Carnobacterium divergens* M35 culture supernatant **(A)**, eluate from SP-Sepharose column **(B)**, eluate from Sep-Pack C18 column **(C)** and purified divergicin M35 **(D)**.

The antimicrobial activity of divergicin M35 during the steps of purification was confirmed using the agar diffusion test (Figure 1). A clear zone of inhibition of *L. innocua* HPB 13 was obtained around the well, confirming anti-listerial activity.

**FIGURE 2:**
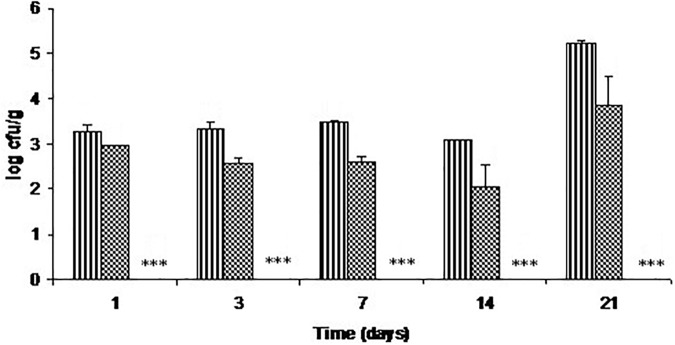
Growth of *L. monocytogenes* on cold-smoked wild salmon during storage at 4°C for 14 days then at 8°C for 7 days: stripe pattern represents untreated fish, checker pattern represents treatment with C-M35 solution; values for the C-M35 film treatment (^∗∗∗^) were below the detection limit (<50 cfu/g).

Figure 2 represents the antimicrobial activity of C-M35 solution and film against *L. monocytogenes* in fish flesh stored at refrigerator temperatures. By day 3, the viable count was about 1 log lower in fish coated with inhibitor solution than in the control, and this difference increased only slightly throughout the storage. There was no reduction in viable count after 14 days of storage. However, the film reduced the count to below the detection limit (<50 cfu/g) from the 1st day of storage.

**FIGURE 3:**
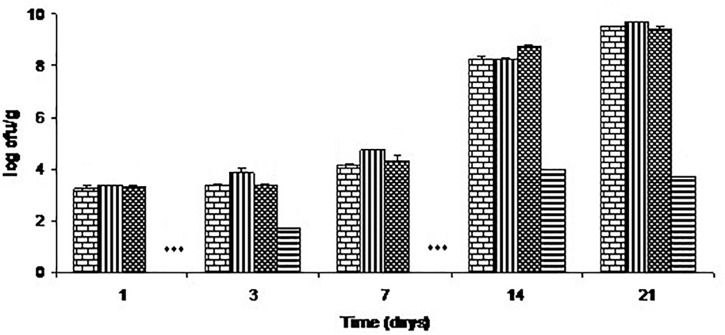
Growth of total aerobes on cold-smoked wild salmon during storage at 4°C for 14 days then at 8°C for 7 days: brick pattern represents the control, striped represents fish inoculated with *L. monocytogenes*, tight weave represents the C-M35 solution treatment and horizontal stripe represents the C-M35 film treatment. ^∗∗∗^ Values were below the detection limit (<50 cfu/g).

Total aerobic counts on these samples are shown in Figure 3. The chitosan-divergicin M35 solution had no effect on the total aerobic count. In contrast, the film had a strong effect, differing from the control by 1.8 log and 5.8 log cycles, respectively, on days 3 and 21.

**FIGURE 4:**
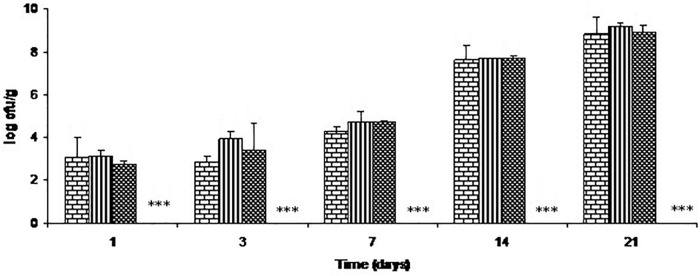
Growth of lactic acid bacteria on cold-smoked wild salmon during storage at 4°C for 14 days then at 8°C for 7 days: brick pattern represents the control, stripe represents fish inoculated with *L. monocytogenes*, tight weave represents treatment with C-M35 solution; values for fish covered with C-M35 film (^∗∗∗^) were below the detection limit (<50 cfu/g).

The effects of C-M35 solution and film on total lactic acid bacteria are illustrated in Figure 4. For all treatments except film, the total lactic acid bacteria counts increased progressively during the 21 days of storage, while counts were below the detection limit (<50 cfu/g) throughout storage with the film.

Counts of *L. monocytogenes*, total aerobes and lactic acid bacteria in the film homogenate were below the detection limit (<50 cfu/g), indicating that no detectable viable cells remained attached to the film (data not shown).

### Residual Antimicrobial Activity in Fish and Chitosan Films

Residual inhibitory activity in the fish treated with C-M35 solution and film and in the C-M35 film after storage is shown in Table 1. For the entire 21-day period, no activity was obtained from fish treated with solution. However, residual activity was detected in fish treated with the film, decreasing significantly over days 1–14 and ending up at 2.3 × 10^3^ on day 21. A residual activity was observed in the film removed after 1 day of storage, decreasing progressively over 14 days and then stabilizing at 4.61 × 10^3^ through to day 21. Fish treated with chitosan solution or film without bacteriocin contained no inhibitory activity, confirming that the residual activity detected was due to divergicin M35 (results not shown).

**Table 1:** Residual divergicin activity (arbitrary units) in homogenate of cold-smoked wild salmon and its chitosan-divergicin M35 film covering after 14 days at 4°C followed by 7 days at 8°C, determined by the agar diffusion test (a) and the critical dilution micro-method (b).

Day	1		3		7		14		21	
					
Method	a	b	a	b	a	b	a	b	a	b
1	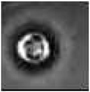	4.61 × 10^3a^	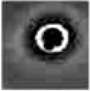	2.30 × 10^3c^	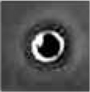	2.30 × 10^3c^	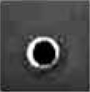	5.76 × 10^2b^	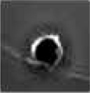	2.30 × 10^3c^
2	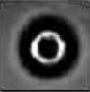	3.69 × 10^4a^	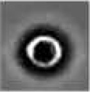	1.84 × 10^4b^	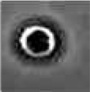	9.22 × 10^3c^	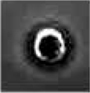	4.61 × 10^3d^	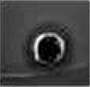	4.61 × 10^3d^
3	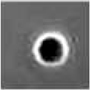	0	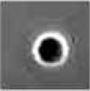	0	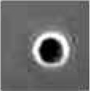	0	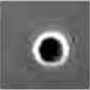	0	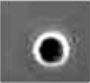	0


*1: In fish treated with chitosan-divergicin film. 2: In chitosan-divergicin film. 3: In fish treated with chitosan-divergicin solution*. *Means with different letters within the same row are significantly different (P < 0.05)*.

### Physical and Chemical Analysis of Fish Samples

**FIGURE 5:**
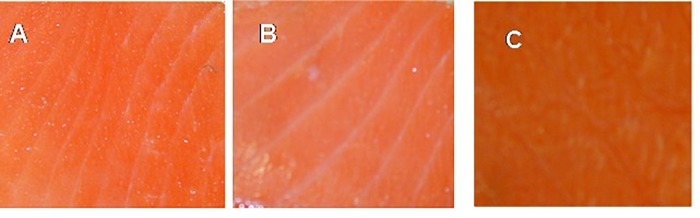
Appearance of cold-smoked wild salmon flesh at the end of 21 days of storage (14 days at 4°C then 7 days at 8°C): control **(A)**, coated with C-M35 solution **(B)**, covered with C-M35 film **(C)**.

The effect of C-M35 solution and film on the color values of un-inoculated fish is shown in Table 2. Differences in lightness (L) between the control and the treatment with solution were significant after 14 days of storage, although no significant difference was observed after 21 days. Application of the film did not produce any significant difference in the *L*-value during the 1st week, although a significant increase was observed thereafter. The redness (a) values of control and solution-treated fish were the same after 1 week but were significantly different after 14 days of storage. For fish treated with film, the redness values were higher than those of the control. The C-M35 solution and film, respectively, resulted in decreased and increased yellowness (b) compared to the control until 21 days of storage. Figure 5 shows the overall appearance and difference in color between the treatments after 21 days of storage. The difference in redness matches the measured (a) values.

**Table 2:** Color parameters L, a, and b for cold smoked wild salmon during storage at 4°C for 14 days then 8°C for 7 days, A: control, E: fish coated with chitosan-divergicin solution, F: fish covered with chitosan-divergicin film.

Color parameter	Day	Treatment		
		
		*A*	*E*	*F*
**L**	1	48.01 ± 1.12^hg^	47.01 ± 1.07^h^	48.81 ± 2.37^fg^
	7	49.89 ± 0.91^def^	50.42 ± 0.87^cde^	49.17 ± 2.69^efg^
	14	48.80 ± 1.12^fg^	52.74 ± 0.97^a^	51.31 ± 0.63^bc^
	21	49.86 ± 1.84^def^	50.67 ± 1.15^bcd^	52.05 ± 0.48^ab^
**a**	1	32.99 ± 2.03^bcd^	32.46 ± 1.31^cd^	34.63 ± 1.58^a^
	7	33.07 ± 1.73^bcd^	31.65 ± 1.03^d^	35.15 ± 0.60^a^
	14	34.86 ± 1.82^a^	29.00 ± 1.40^e^	34.15 ± 0.65^ab^
	21	32.95 ± 2.22^bcd^	28.15 ± 2.13^e^	33.86 ± 0.71^abc^
**b**	1	32.38 ± 1.55^drf^	29.71 ± 1.33^g^	34.39 ± 2.27^abc^
	7	34.02 ± 1.12^bc^	31.76 ± 1.31^f^	34.73 ± 2.11^abc^
	14	33.75 ± 1.63^cd^	31.89 ± 1.06^ef^	35.90 ± 1.03^a^
	21	33.43 ± 2.70^cde^	28.83 ± 0.96^g^	35.47 ± 0.82^ab^


*Means ± std with different letters for the same color parameter are significantly different (P < 0.05).*

**FIGURE 6:**
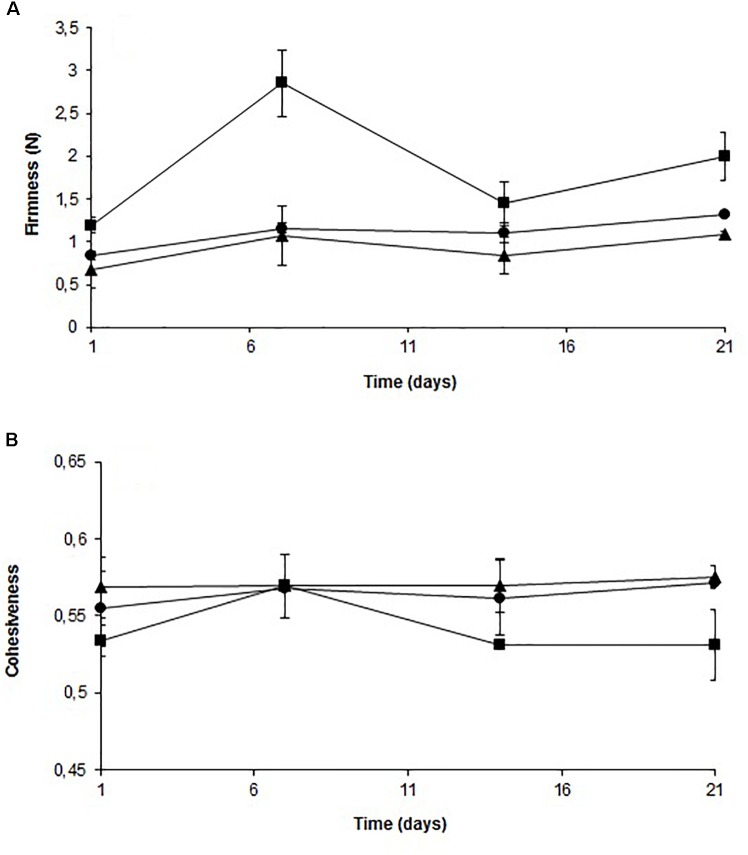
Evolution of firmness **(A)** and cohesiveness **(B)** of cold-smoked wild salmon during storage at 4°C for 14 days followed by 8°C for 7 days: triangle represents control, octagon represents treatment with C-M35 solution and square represents treatment with C-M35 film.

Figure 6 shows the firmness (A) and cohesiveness (B) profiles of the stored fish samples. Firmness increased in samples treated with solution, although no significant difference was observed compared to the control. For fish treated with film, there was a significant difference throughout the storage period in the firmness value compared to the control. The solution did not affect cohesiveness significantly during the storage. The cohesiveness value of fish treated with film was the same after 1 week of storage compared to the control and decreased significantly after 14 days of storage.

The pH of fish treated with C-M35 solution was stable, ranging between 5.9 and 6 during the 21 days of storage, and no significant difference was observed compared to the control (data not shown). Application of the film resulted in a decrease to 5.5 after 1 day of storage and this value was maintained throughout the 3 weeks of storage. TVBN production increased from 10.3 to 30.4 mg N/100 g in solution-treated samples over the 21 days, which was similar to the control range of 12.4–29.9 mg N/100 g (results not shown). Application of the film slowed TVBN production significantly to 17.5 mg N/100 g at the end of the 21 days.

## Discussion

The efficacy of bacteriocins as bio-preservatives is influenced by various food matrix parameters and by the form in which they are applied. One of the most promising strategies proposed to enhance efficacy is incorporation into polymer films. Polymer-based films allow direct contact with the food surface and therefore continuous release of the antimicrobial compound, allowing the maintenance of inhibitory activity on food surfaces (Quintavalla and Vicini, 2002; Ture et al., 2011; Fu et al., 2016).

The combination of a natural polymer such as chitosan (by itself somewhat inhibitory) with a natural soluble inhibitor such as divergicin M35 is a plausible strategy for suppressing bacterial growth in ready-to-eat products and one that may be acceptable to consumers. Our work has shown clearly that this combination does inhibit *L. monocytogenes* on cold-smoked salmon and much more when applied as a film, in which case it can keep viable counts below the detection limit (<50 cfu/g) for 21 days. Since *L. monocytogenes* did not infiltrate the film, we can conclude that inhibition was complete. This was no doubt due to a continuous release of divergicin M35. Previous studies have shown that antimicrobial effects of active films in various food systems depend on the nature and concentration of the antimicrobial agent used. Ye et al. (2008b) increased the anti-*L. monocytogenes* activity of chitosan-coated plastic film on cold-smoked salmon by incorporating sodium lactate alone or with nisin or potassium sorbate. They obtained similar results on ham steaks by incorporating with these compounds as well as sodium diacetate or sodium benzoate Ye et al. (2008a). Sodium lactate was the most effective in this case. Marcos et al. (2007) found that alginate was superior to zein and polyvinyl alcohol as an enhancer of *L. monocytogens* inhibition by enterocin at 200 or 2,000 units per cm^2^.

The weak anti-listerial activity and absence of residual activity of the C-M35 solution in the sample homogenate is likely due to interactions between the bacteriocin and food components such as lipids as well as partial inactivation by endogenous proteases produced *in situ* by other microorganisms (Katla et al., 2001; Vaz-Velho et al., 2005). Aasen et al. (2003) showed that more than 80% of the sakacin P added to cold-smoked salmon and chicken was adsorbed to proteins. The antimicrobial activity of inhibitory substances in cold-smoked salmon may also be affected by added salt. Devlieghere et al. (2004) observed that adding NaCl to a medium decreased the antimicrobial activity of chitosan, due to interaction between Cl^-^ ions and positive charges on the chitosan and to competition of Na^+^ for the negative charges on the microbial cell surface, and that antimicrobial activity also depended on pH and the isoelectric point of proteins in the food matrix. These phenomena probably affect bacteriocin activity, whether free or in film. However, continuous release of divergicin M35 during storage may minimize such losses. Our results showed that the fish and the film covering contained residual activity of divergicin M35 during the 21 days of storage. The difference in activity added and activity recovered in the film and fish is probably due to the phenomena mentioned above.

The total aerobic and lactic acid bacteria counts as well as TVBN production suggest that the antimicrobial solution was practically ineffective while the antibacterial film was quite effective. Since initial levels of bacteria in this salmon product were low, the bacteriocin-loaded chitosan film reduced these to below the limit of detection. Ye et al. (2008a) also obtained a substantial reduction in total and anaerobic counts using their chitosan-coated plastic film loaded with antimicrobial agents. The microbiological results obtained in our study demonstrate the potential of divergicin-loaded chitosan film for extending the shelf life of cold-smoked salmon. Previous reports that chitosan inhibits the growth of a wide variety of microorganisms (Shahidi et al., 1999; Jeon et al., 2002; Agullo et al., 2003; Rinaudo, 2006; Yingyuad et al., 2006). However, and to the best of our knowledge, no study has been published on application of chitosan based film containing bacteriocin to improve the preservation of cold-smoked salmon during storage.

In the present study, we were also interested in the impact of chitosan-divergicin M35 film on the physical and chemical characteristics of the fish product. The increase in firmness during the 1st week of storage was likely due to moisture loss, while changes during the 2nd week could be due to proteases produced by spoilage microorganisms (Morzel et al., 1997) or to autolytic enzymes in the fish flesh (Stoknes and Rustad, 1995). The significant differences obtained with film were probably related to the strong affinity of chitosan NH_2_ groups for water molecules (Wu et al., 2004). The increase in the firmness of film-covered fish during the last week of storage may be due to the onset of film breakdown. The cohesiveness of these samples decreased slightly during the last week of storage, which was consistent with the changes in the muscle strength retention capacity after the first compression.

Increased brightness (L) and decreased redness and yellowness (a and b) values of C-M35 solution-treated compared to untreated fish are consistent with previous observations. Jo et al. (2001) observed similar changes in brightness and redness for sausage containing chitosan as a preservative. Tahiri et al. (2009) reported a significant decrease of the yellowness value of divergicin-M35-treated cold-smoked salmon stored for 21 days at 4°C. The increased in redness of film-covered samples is probably due to decreased breakdown of carotenoids because of the low oxygen permeability of chitosan film (Xu et al., 2005). The loss of water from the sample may also result in increased pigment concentration (Choubert et al., 1992).

For all treatments, the TVBN concentration was below the acceptable maximum proposed by various authors (Civera et al., 1995; Leroi et al., 2001). The C-M35film-covered samples thus showed a higher hygienic quality compared to the control and solution-treated samples. TVBN concentration is considered a major quality index for cold-smoked salmon (Leroi et al., 2001; Brillet et al., 2005). These low concentrations could be a result of reduced counts of spoilage bacteria due the effect of the C-M35 film. Jeon et al. (2002) reported reductions in TVBN of 33–50% and 26–51% in chitosan-glycerol-coated cod and herring, respectively, at the end of 12 days of storage at 4°C.

## Conclusion

In this study, the combination of chitosan and divergicin M35 was more effective for inhibiting *L. monocytogenes* in cold-smoked wild salmon when applied as a film rather than as a solution. The film reduced *L. monocytogenes* to below detection limit (<50 cfu/g). The film was shown to maintain acceptable color and texture of this fish product during 3 weeks of storage at refrigerator temperatures. This represents an extension of product shelf life. Additional studies using sensory evaluation panels should be conducted before considering commercial application of such films.

## Author Contributions

RB was the Ph.D. student in charge of performing all the laboratory work. RB also was in charge of the preparation of the manuscript. MS was in charge of the laboratory work related to the preparation and characterization of divergicin-chitosan films. MD was involved in the preparation and study of the antimicrobial activity of divergicin M35 and chitosan. IF supervised the entire work and was in charge of the microbiological aspects of the work.

## Conflict of Interest Statement

The authors declare that the research was conducted in the absence of any commercial or financial relationships that could be construed as a potential conflict of interest.
